# Biomarkers of acute lung injury: worth their salt?

**DOI:** 10.1186/1741-7015-9-132

**Published:** 2011-12-12

**Authors:** Alastair G Proudfoot, Matthew Hind, Mark JD Griffiths

**Affiliations:** 1 Royal Brompton & Harefield NHS Foundation Trust, Adult Intensive Care Unit, Sydney Street, London SW3 6NP, UK; 2 Unit of Critical Care, National Heart and Lung Institute, Imperial College London, London SW3 6LY, UK

## Abstract

The validation of biomarkers has become a key goal of translational biomedical research. The purpose of this article is to discuss the role of biomarkers in the management of acute lung injury (ALI) and related research. Biomarkers should be sensitive and specific indicators of clinically important processes and should change in a relevant timeframe to affect recruitment to trials or clinical management. We do not believe that they necessarily need to reflect pathogenic processes. We critically examined current strategies used to identify biomarkers and which, owing to expedience, have been dominated by reanalysis of blood derived markers from large multicenter Phase 3 studies. Combining new and existing validated biomarkers with physiological and other data may add predictive power and facilitate the development of important aids to research and therapy.

## Introduction

The syndrome acute lung injury (ALI) and its more severe counterpart acute respiratory distress syndrome (ARDS) are defined by radiographic and physiological changes that characterize patients with acute lung failure (Table 1) [1]. All age groups may be affected, although the syndrome has a higher incidence and mortality in older people. Across all ages the incidence is approximately 200,000 cases per year in the United States with a mortality of around 35% [2]. Survivors face a long-term reduction in quality of life; for example, only 54% of survivors were able to return to work 12 months after hospital discharge [3].

**Table 1:** NAECC definition of Acute Lung Injury (ALI) and Acute Respiratory Distress Syndrome (ARDS)[1]

	Timing	Oxygenation	Chest Radiograph	Exclusion of cardiogenic pulmonary oedema
**ALI** **ARDS**	Acute	PaO_2_/FiO2 ≤ 300 mmHg or 40 kPa PaO_2_/FiO_2 _≤ 200 mmHg or 27 kPa	Bilateral opacities consistent with pulmonary oedema	PAOP ≤ 18 mmHg if measured or no clinical evidence of left atrial hypertension
				

PaO_2_/FiO_2_, arterial partial pressure of oxygen/inspired oxygen fraction; PAOP, pulmonary artery occlusion pressure

The validation of biomarkers, for use in clinical trials and ultimately in practice, has become a central tenet of translational biomedical research [4]. The purpose of this article is to discuss the role of biomarkers in the management of ALI and related research. We shall not present a state of the art review of the field of all the biomarkers that have been investigated in this field, excellent examples of which have been produced recently [5,6]. Rather, we shall question current strategies to identify biomarkers and whether what has been achieved thus far has advanced the field.

## The natural history of acute lung injury

Regardless of the wide variety of insults that cause or contribute to the development of ALI, the response of the lung is largely stereotypic. A combination of tissue injury and inflammation affecting the gas exchange surface of the lung, the alveolar-capillary membrane, causes high permeability pulmonary edema. The presence of a protein-rich inflammatory exudate in the airspace impairs surfactant function [7]. The resulting collapse and consolidation of the lung causes profound hypoxemia because inflammatory mediators induce changes in the control of vascular tone that disable hypoxic pulmonary vasoconstriction [8]. Loss of pulmonary capillary surface area associated with localized lung destruction and occlusion of the vascular bed by intravascular thrombosis, increases the anatomical dead space, itself associated with a poor outcome [9], giving rise to carbon dioxide retention. Host factors, both inherited [10,11] and acquired, influence individual susceptibility, (for example, excessive alcohol consumption predisposes, while diabetes mellitus protects) [12,13]. Precipitating causes or risk factors, which often "hunt in packs", either affect the lung directly (pneumonia, aspiration of stomach contents and thoracic trauma) or cause ALI indirectly through a systemic inflammatory response syndrome (SIRS) associated with multiple organ dysfunction, exemplified by severe sepsis and transfusion related ALI [14]. These causes, to a large part, determine the initial clinical course and outcome, but most patients subsequently require invasive mechanical ventilation in an intensive care unit to maintain adequate gas exchange and often other organ supports.

While the development of pulmonary fibrosis in a patient with ALI predicts the requirement for prolonged respiratory support and a poor outcome [15], relatively little is known about the processes that determine the resolution of inflammation, injury and subsequent lung repair [16]. The consecutive three-phase pathological model of ALI (exudative, proliferative and fibrotic) is a gross over-simplification. Fibrosis is evident histologically as early as a week after the onset of the disorder [17] and procollagen III peptide, a precursor of collagen synthesis, is elevated in the broncho-alveolar lavage (BAL) fluid of ARDS patients at the time of tracheal intubation [18]. Indeed, not only is the injured lung known to be heterogeneously affected [19], it also seems likely from the examination of lavage samples from patients that these pathological processes coincide in the same lung region [20]. Similarly, while several pro-inflammatory mediators are also pro-fibrotic, distinct patterns of gene expression are associated with acute inflammation and fibrosis in the injured lung, suggesting that fibrosis is not simply an inevitable consequence of unresolved inflammation [21]. Indeed, current thinking emphasizes the primary role of disordered epithelial repair, which may be contributed to by repeated or persistent injury and inflammation, in driving a pathological fibrotic response [22].

**Figure 1:**
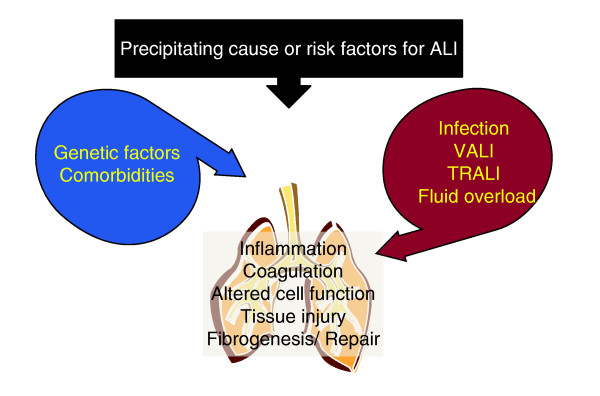
**Process-based pathogenesis of ALI**. Traditional causes of or risk factors for ALI maybe contributed to in certain patients by hospital-acquired harm (red) and modified in their potency for causing ALI by multiple patient's susceptibility (blue). Depending on the balance of these factors the processes that determine the natural history of ALI are initiated in the lung.

Despite years of concerted effort and very many clinical trials, a minority of which have been capable of producing a definitive result, there are no treatments (as opposed to modifications of organ support [23,24]) that improve the outcome of patients with ALI [25]. What has become evident, both in this field and in critical care in general, is the extent and importance of iatrogenic injury. Hence, half of ALI arises in patients who were subjected to mechanical ventilation for another reason: the four major culprits being mechanical ventilation that targets normal blood gas parameters, transfusion of blood products, excessive fluid resuscitation and hospital acquired pneumonia (Figure 1) [26-29]. Accordingly, recent epidemiological evidence suggests that targeting hospital acquired injury can halve the incidence of ARDS despite an increase in patients' severity of illness, the number of comorbidities and the prevalence of major ARDS risk factors [30].

Hence, studying patients with ALI is a challenge because the syndrome is the end result of an almost infinite variety of scenarios. These range from young fit patients with severe pneumonia or thoracic trauma to older patients who fail to recover from routine procedures, suffer complications, require respiratory support because of a combination of a chronic cardio-respiratory condition and hospital-acquired pneumonia, and ultimately develop ARDS on a ventilator. As a consequence, the water is muddied both by heterogeneity in the host and in the risk factors, and by the variety of other co-incident processes. Furthermore, it is often difficult to define precisely when the syndrome started, which may have a dramatic effect on measured variables in cases where the condition changes rapidly. Finally, variable management regimens may contribute to patient heterogeneity, both in the face of clear evidence (for example, poor adherence to low tidal volume ventilation) [31] and where evidence is lacking (for example, in the use of adjuncts to respiratory support like prone positioning, inhaled nitric oxide and high frequency oscillation). Conversely, critically ill patients are closely monitored, physiological data are electronically stored and their clinical condition may paradoxically render them more amenable to undergoing invasive procedures.

## Why invest in biomarkers for ALI?

Biomarkers are potentially useful as guides to clinical management and as research tools. In the clinical setting there is a high premium on biomarker data being easily and safely obtained within a timeframe that is relevant to the disease process (Table 2). For example, an indicator of poor prognosis that may encourage referral to a specialist center would need to be available within hours, whereas a marker of ventilator-associated lung injury (VALI) that was being used to fine-tune ventilator settings would have to be "turned around" within minutes. No biomarkers that are currently available have penetrated routine clinical practice with the possible exception of the use of procalcitonin (PCT) to diagnose sepsis in critically ill patients and to guide their antibiotic therapy [32]. Sepsis syndromes commonly both cause and complicate ALI; ventilator associated pneumonia in particular frequently exacerbates ALI causing diagnostic difficulty. Procalcitonin levels correlated with severe sepsis and bacteraemia [33], but did not consistently differentiate survivors from non-survivors [34]. A PCT-based algorithm guiding initiation and duration of antibiotic therapy in critically ill patients with suspected bacterial infection was associated with a 23% relative reduction in antibiotic exposure with no significant increase in mortality [32]. Aside from this role in limiting antibiotic exposure, a recent review of the role of PCT in diagnosing ventilator associated pneumonia concluded that the biomarker showed good specificity but low sensitivity [35].

**Table 2:** Proposed characteristics of an ideal biomarker for acute lung injury

Measurement is safe and feasible in the critically ill	
Sensitive, reproducible and specific	
Timely	
Modified by an effective intervention to change the target outcome of interest	

Clinical research in ALI has used biomarkers as surrogate outcomes for early Phase 2 studies and may in the future be valuable in categorizing patients into subgroups that are predicted to be most likely to benefit from particular interventions. For example, in the single-centre BALTI 1 study 40 patients with ALI were enrolled to demonstrate the ability of seven days of treatment with intravenous salbutamol to decrease extravascular lung water measured by the single indicator transpulmonary thermodilution method (PiCCO, Pulsion Medical Systems, Munich, Germany) [36]. The resolution of pulmonary edema is central to recovery from ALI as it entails defervescence of air space inflammation and restoration of a functioning alveolar-capillary membrane. Accordingly, elevated extravascular lung water measured using this technique early in the course of ALI/ARDS, particularly if indexed to predicted body weight, was associated with a poor outcome [37-39]. The initial ratio of PaO_2_/FiO_2 _was reported to be lower in non-survivors [9,40-42] and predicted mortality in univariate analyses [9,40,42]. In addition, in one large cohort study, PaO_2_/FiO_2 _ratio was an independent predictor of mortality [42]. However, this variable does not take into account the mode or even presence of mechanical ventilation and apart from measurements at the extremes of the spectrum, it is generally not considered to be a robust predictor of outcome in ALI. Hence, the use of a surrogate rather than a clinical end-point, such as ventilator-free days or intensive care unit length of stay, decreased the recruitment target to what was achievable for a single center. However, in hindsight it is arguable whether this positive result then justified the investment in two Phase 3 large multicenter trials, which failed to show a survival benefit from using both inhaled and intravenous formulations of short-acting beta agonists [25].

The use of biomarkers to refine patient populations, such that clinical trials will be most likely to provide a definitive answer requiring the fewest patients, is particularly appealing for application to research involving patients with a heterogeneous syndrome like ALI. This could be beneficial by helping to characterize either a group of patients with a high mortality where mortality is the primary outcome measure, or by identifying patients in whom a pathological process, which is targeted by an intervention, is particularly prominent.

## Characteristics of ideal biomarkers for ALI

Proposed criteria for characterizing ideal biomarkers for ALI, most of which are self-explanatory are listed in Table 2. It has been argued that biomarkers should inform or, at least, relate to the disease pathogenesis [43]. We disagree for philosophical reasons. Why confuse elucidating mechanisms with the pragmatic business of identifying biomarkers? We prefer as wide a definition as possible; for example, the electrocardiograph has been one of the most useful biomarkers in medicine but not much can be learned about the pathogenesis of myocardial infarction through its study.

The current definition of ALI/ARDS is such that biomarkers of the established syndrome are largely redundant. An exception would be a biomarker that was specific to the pathological process described as diffuse alveolar damage. That is, a biomarker that could exclude patients from studies who fulfilled the diagnostic criteria but who essentially have a distinct disease, which may have a different natural history and specific treatment, for example, cardiogenic pulmonary oedema, eosinophilic pneumonia and pulmonary embolism. Most studies have attempted to correlate selected biomarkers with disease severity or death, which is potentially useful both clinically to help target resources and more expensive or invasive management strategies, and in helping to power research studies using mortality as the primary outcome.

We propose that the use of biomarkers in a complex syndrome like ALI is most likely to be effective when they are specific to an individual component or process that can be manipulated. One productive approach has been to measure plasma and BAL fluid levels of mediators as a reflection of systemic and pulmonary inflammation respectively. In samples from large multicenter trials elevated levels of mediators, like soluble tumor necrosis factor-alpha receptors (sTNFR) 1 and 2 [44], soluble intercellular adhesion molecule-1 [45], and interleukin (IL)-6 [23] were associated with adverse outcomes in patients with ALI. The limitations of this strategy are that these mediators have multiple effects, have no specificity to the lung and there is no convincing evidence that manipulating the inflammatory response benefits patients with ALI. Partly because of the realization that VALI plays a major part in the pathogenesis of ALI and, as a result, many large studies have been performed examining the effects of ventilator strategies, a lot has been learned about the responses of popular biomarkers in patients undergoing protective and standard ventilation [6]. Hence, circulating mediators of inflammation (sTNFR [45], IL-6, -8 and -10 [46]), indicators of epithelial cell injury (soluble advanced glycation end-product receptors (sRAGE)) [47] and surfactant protein-D [48]) and components of the coagulation system (protein-C and plasminogen activator inhibitor-1 [49]) have all been promoted as biomarkers of VALI. However, because the proposed mechanism, whereby VALI kills patients through the exacerbation of local injury and inflammation, the mediators of which then leak into the systemic circulation causing multiple organ dysfunction [50], it would be surprising if there was not considerable overlap between markers of VALI, tissue injury, inflammation and a poor prognosis. In other words, these biomarkers inevitably lack specificity for individual processes or outcomes.

More recently the power of combining clinical parameters with a panel of traditional biomarkers to predict mortality in patients with ALI using a variety of statistical techniques has been examined in the large datasets and sample stores resulting from ARDS Network studies [51,52]. In one of these studies [52], the six clinical predictors were: age, the underlying cause, APACHE III score, plateau pressure, number of organ failures, and alveolar-arterial difference in the partial pressure of oxygen measured at enrollment prior to randomization. Eight biomarkers were measured in baseline plasma samples from enrolled patients that reflected endothelial and epithelial injury, inflammation and coagulation. A "reduced model", including just the APACHE III score, age, SP-D, and IL-8, performed almost as well as that which included all parameters and biomarkers. However, the additional predictive value of the plasma biomarkers added to the clinical predictors alone was modest; thus, further work will be needed to test the value of these biomarkers over clinical predictors alone. Furthermore, while the inclusion of biomarker data into a model improved the accuracy of mortality prediction, the predicted risk of death for the patients who ultimately died remained lower than 50%, suggesting that important contributors to mortality may not have been accounted for by the model [51].

## Model systems for biomarker development

**Figure 2:**
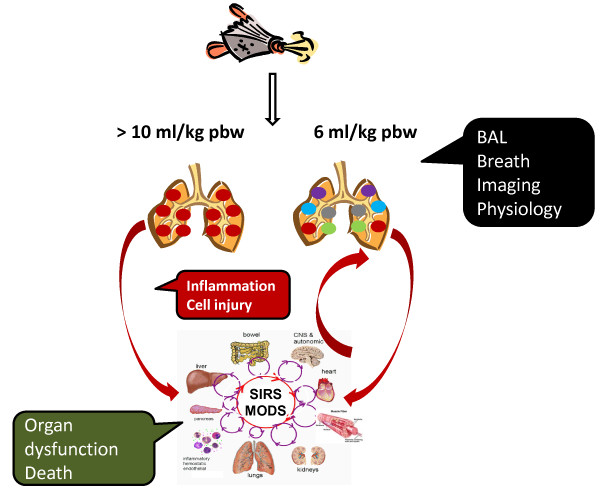
**ALI biomarkers: Ventilator Induced Lung Injury experimental models and observational clinical studies**. High tidal volume ventilation (tidal volume in excess of 10 ml/kg predicted body weight) may be used to induce lung injury in experimental models (left) but, through the overspill of inflammatory mediators into the circulation (biotrauma), multiple-organ dysfunction may follow. Biomarkers may be assayed directly from the lungs (black), from the circulation (red) or as indices of dysfunction related to other organs (green). In clinical studies (right), injured lungs are susceptible to damage even when gold standard mechanical ventilation (tidal volume 6 ml/kg predicted body weight) is used. However, in the presence of existing lung injury several processes are likely to be concurrent, both affecting the lungs directly (inflammation, tissue injury, coagulation, fibrosis) and indirectly from other affected organs (sepsis). Hence, the relationship between any biomarker and ventilator settings is likely to be obscured by multiple unknowns. BAL bronchoalveolar lavage, SIRS systemic inflammatory response syndrome, MODS multiple organ dysfunction syndrome.

An alternative approach to examining clinical samples is to test the validity of existing or novel candidate biomarkers using models systems, in which the signal-to-noise ratio is likely to be more favorable and the time course of the biomarker's response can be more precisely determined (Figure 2). For this purpose, we believe that human models are likely to be more useful than animal models, despite the undoubted contribution of the latter to our understanding of the syndrome's pathogenesis [53,54]. For example, at the most basic level comparative proteomic analysis between BAL fluid from a patient and a mouse model of ALI identified only 21 homologous proteins [55].

For an example of a human model of ALI, one lung ventilation (OLV), a technique required to facilitate lung resection surgery, has been exploited to investigate potential biomarkers of VALI [56,57]. One-lung ventilation may be a useful model of VALI because it is associated with a smaller lung volume available for ventilation, localized lung collapse or atelectasis and impaired oxygenation, resulting in exposure of the ventilated lung to volutrauma, repeated opening of collapsed airspaces (atelectotrauma), and a high inspired oxygen concentration. High tidal volume and airway pressure during OLV correlated with the development of ALI in patients undergoing lung resection {Fernandez-Perez, 2009 #1309 [58]; Licker, 2003 #38; Jeon, 2009 #532} and the incidence of ALI after lung resection over a five-year period was lower, compared to an historical control group, after introduction of a protective OLV protocol [59]. In small prospective studies the use of low tidal volume OLV was associated with reduced biomarkers of pulmonary and systemic inflammation {Michelet, 2006 #4882; [60]} Finally, in an observational prospective study of 30 patients, exhaled breath condensate pH was reduced within minutes of starting OLV suggesting that it may represent a robust and direct means of sampling the milieu of the lung. While, in the clinical setting the effect on exhaled breath condensate pH of changing ventilator settings may be drowned by "noise" from co-incident inflammatory processes in the lung, it may hold promise as a non-invasive real-time biomarker of VALI, despite the fact that the mechanism by which exhaled breath condensate acidification is poorly understood (Figure 2).

## Future directions

Despite analyzing samples from large well-designed trials, there is no biomarker in current use that positively identifies patients with the classical histopathological appearances of diffuse alveolar damage, that predicts a poor outcome or that specifically identifies a pathological process [61].

We propose that future biomarker development be driven by novel therapies and support modalities. Biomarkers potentially combined with physiological and genomic data should be used to identify patient groups for research studies and individuals who are most likely to gain from targeted therapies. For example, novel extra-corporeal carbon dioxide removal (ECCO_2_R) systems that will make these techniques safer, cheaper and more readily available should stimulate the search for novel biomarkers for VALI. The demonstration that gold standard low tidal volume protective ventilation was associated with signs of over-distension on CT scanning, elevated plasma markers of inflammation and a plateau airway pressure greater than 28 cmH_2_O in approximately a third of patients with ARDS provided a readily identifiable population on which the effectiveness of novel ECCO_2_R devices could be tested [62]. In a subsequent study, a group of patients with ARDS was identified by their having a plateau airway pressure greater than 28 cmH_2_O and in whom low tidal volume ventilation was presumed to be causing significant VALI. For these patients a novel ECCO_2_R device (DeCap, Hemodec, Salerno, Italy) enabled the research team to decrease tidal volume, further targeting a plateau airway pressure of less than 25 cmH_2_O, which was associated with a lower radiographic index of lung injury and lower levels of lung derived inflammatory cytokines [63].

Targeting component processes of ALI other than inflammation, tissue injury and VALI should be developed and incentive for this will be greatly increased as novel therapeutics are introduced (Figure 1). We believe that advances in the understanding and treatment of pulmonary fibrosis combined with the relative chronicity of this process complicating ALI, which leads to patients with a low pulmonary compliance struggling to wean from ventilatory support, make this process suitable for targeted intervention. For example, the significance of the epithelial cell integrin alpha-v beta-6 in activating transforming growth factor beta-1 (TGFβ-1) both in the evolution of ALI and repair by fibrosis has only recently been appreciated [64,65]. Supposing an effective means of targeting this pathway existed, how could the decision to administer a novel therapy be informed using biomarkers? Patients who are susceptible to or have over-activity of this pathway may be identified using a genomic approach or by identifying a biomarker that is either specific to this process or a damaging fibrotic response. Indeed, the feasibility of this approach has been illustrated by the observation that elevated levels of procollagen peptide III in lavage fluid from patients on Day 3 of ARDS were independent risk factors for mortality [66]. This so-called personalized approach has been pioneered in other chronic lung diseases but the principles are theoretically applicable to ALI [67].

## Conclusions

Biomarker development for patients with ALI is an essential component of progress in translational medicine in this challenging area. Potential pitfalls on the road to successfully carrying out translational medicine to help patients with ALI include:

1. the heterogeneity of cases (ALI is a syndrome resulting from any cause of acute lung failure),

2. the large iatrogenic contribution to pathogenesis, rendering standardization of care crucial,

3. the gas exchange surface of the lung is relatively inaccessible to investigation and indispensible, so that pathogenic process are not well understood, and

4. the timeframes of the condition are short, such that windows for intervention may close in the time that it takes to analyse samples and process data.

Biomarkers should be sensitive and specific indicators of clinically important processes and should change in a relevant timeframe to affect recruitment to trials or clinical management. They do not necessarily need to reflect pathogenic processes. Combining biomarkers with physiological and other data may add predictive power and stimulate the development of important aids to research and therapy. While biomarkers have not yet had a major role in the management of ALI and the development of novel therapies, it is possible and even likely that biomarkers will be developed that will help to target an increasing arsenal of disease modifying therapies in the future.

## Abbreviations

ALI: acute lung injury; ARDS: Acute Respiratory Distress Syndrome; BAL: broncho-alveolar lavage; ECCO_2_R: extra-corporeal carbon dioxide removal; OLV: one lung ventilation; PCT: procalcitonin; SIRS: Systemic Inflammatory Response Syndrome; sTNFR: soluble tumour necrosis factor-alpha receptors; TGFβ-1: transforming growth factor beta-1; VALI: ventilator associated lung injury.

## Competing interests

MG received consultancy fees, educational grants and served on advisory boards for GlaxoSmithKline (Middlesex, UK). AP and MH have not disclosed any potential conflicts of interest.

## Authors' contributions

All authors contributed equally to the inception and execution of this article and read and approved the final manuscript.

## Pre-publication history

The pre-publication history for this paper can be accessed here:

http://www.biomedcentral.com/1741-7015/9/132/prepub
